# Regenerating island-derived protein 3E concentrations in healthy dogs and dogs with various inflammatory processes

**DOI:** 10.1371/journal.pone.0324090

**Published:** 2025-05-20

**Authors:** Laureen M. Peters, Theresia Reding Graf, Rolf Graf, Meike Mevissen, Judith Howard

**Affiliations:** 1 Department of Clinical Veterinary Medicine, Clinical Diagnostic Laboratory, Vetsuisse Faculty, University of Bern, Bern, Switzerland; 2 Department of Surgery and Transplantation, Pancreas Research Laboratory, University Hospital, University of Zürich, Zürich, Switzerland; 3 Department of Clinical Research and Veterinary Public Health, Division of Veterinary Pharmacology and Toxicology, Vetsuisse Faculty, University of Bern, Bern, Switzerland; University of Life Sciences in Lublin, POLAND

## Abstract

**Background:**

Blood concentrations of regenerating island-derived proteins (REG) are high in humans with sepsis, acute pancreatitis, and gastrointestinal diseases. REG3E was recently identified in canine pancreas and blood, but concentrations in dogs with various diseases are unknown.

**Objectives:**

To measure and compare REG3E concentrations in dogs with sepsis, acute pancreatitis, gastrointestinal diseases, and healthy dogs.

**Methods:**

Cross-sectional study measuring plasma REG3E concentrations using an in-house developed ELISA in stored convenience samples from client-owned dogs with naturally occurring sepsis (n = 44), acute pancreatitis (n = 42), acute (n = 25) and chronic gastrointestinal diseases (n = 23), and healthy controls (n = 80). Concentrations of REG3E are compared among groups and between survivors and non-survivors to discharge using the Kruskal-Wallis test with post-hoc Conover analysis and independent samples t-tests.

**Results:**

REG3E concentrations differed significantly among all groups (*P* < 0.005), except between sepsis and acute pancreatitis (*P* = 0.936). Median concentrations (interquartile range [ng/mL]) were 36.5 (30–89) in healthy dogs, 200 (103.5–361.5) in dogs with chronic gastrointestinal diseases, 634 (257–1060) in dogs with acute gastrointestinal diseases, 1644.5 (710 − 4122) in dogs with acute pancreatitis, and 1736 (480.5–3416) in septic dogs. Non-survivors (n = 33) had significantly higher REG3E concentrations than survivors when compared across all clinically ill dogs (*P* < 0.001) and within the sepsis group (n = 20; *P* = 0.0133), but not within the acute pancreatitis group (n = 13; *P* = 0.248).

**Conclusions and clinical importance:**

Plasma REG3E concentrations are higher in dogs of all disease categories, particularly in dogs with sepsis and acute pancreatitis, but with considerable overlap between different diseases. High REG3E concentrations may be associated with poor prognosis in septic dogs. Thus, REG3E is likely to reflect inflammation and merits further investigations to refine its potential as a diagnostic and decision-making biomarker in this species.

## Introduction

Infectious diseases can lead to systemic inflammatory response syndrome (SIRS) and sepsis, with a potentially fatal outcome if not diagnosed and promptly treated [1]. Leukogram parameters, such as neutrophilia, neutropenia, left shift and toxicity, as well as neutrophil-to-lymphocyte ratios provide insight into the general inflammatory state of the animal but have not proven useful to distinguish between sepsis and non-septic SIRS nor to reliably predict outcome [2,3]. Upregulation of the pro-inflammatory cytokines TNF-α, IL-1, and IL-6 stimulates the production of positive acute-phase proteins such as c-reactive protein (CRP) [4], the concentration of which increases reliably in inflammation in dogs, but is nonspecific and unable to reliably distinguish between sepsis and non-septic SIRS, nor to provide an accurate prognosis [5,6]. Procalcitonin (PCT), a thoroughly investigated biomarker for the diagnosis and prognosis of bacterial infection in humans [7], has shown promise as a novel biomarker for sepsis in dogs after it was demonstrated to significantly increase after injection of lipopolysaccharide in healthy dogs [8]. Concentrations of PCT were higher in dogs with naturally occurring sepsis compared with healthy dogs, and higher in dogs with septic shock compared with sepsis without cardiovascular compromise [9,10]. However, there was considerable overlap between these groups, and another study has found no significant increase of PCT concentrations above reference values despite confirmed bacterial infection [11], limiting the use of PCT as the sole biomarker for the diagnosis and stratification of sepsis in dogs. As sepsis is a ubiquitous disorder with a non-specific clinical picture, associated with high mortality, more accurate and reliable biomarkers for its diagnosis and prognosis are desirable.

Pancreatic stone protein, also known as regenerating island-derived (or islet-derived) protein (REG) 1A, a protein primarily produced in the pancreas [12], has been investigated as a novel biomarker for sepsis in humans in the last decade [13]. Concentrations of REG1A can reliably distinguish sepsis from non-septic SIRS, correlate well with disease severity, and can predict development of sepsis and non-survival, performing at least as well as CRP and PCT in several studies [14–16]. Additionally, REG1A and REG3A (also known as pancreatitis-associated protein or PAP) from the same protein family are high in people with pancreatitis, particularly severe acute pancreatitis (AP) [17–19]. Furthermore, REG3A is higher in serum and feces of people with active Crohn’s disease compared with patients in remission and healthy volunteers [20].

In dogs, information on REG proteins is scarce. One study identified REG3E, a homolog of human REG3A, in canine pancreatic tissue and plasma, with higher expression in one case each of acute pancreatitis and acute hemorrhagic diarrhea syndrome with sepsis, but found no evidence of members of the REG1 subfamily in this species [21]. Another study described higher concentrations of REG3A/PAP-1 in feces of dogs with chronic enteropathies compared with healthy controls [22].

Our goals were to compare plasma REG3E concentrations between healthy dogs and dogs with diseases potentially leading to high REG concentrations extrapolated from the human literature, including sepsis, AP, and gastrointestinal diseases (GID), in order to gain preliminary insight as to whether this protein has potential to discriminate among disease groups. An additional aim was to compare REG3E concentrations between survivors and non-survivors, to assess whether this marker could predict outcome, paralleling the prognostic value of REG1A in septic humans. We hypothesized *a priori* that REG3E concentrations are significantly higher in sick compared with healthy dogs, with the highest concentrations expected in dogs with AP, and that non-survivors have higher REG3E concentrations.

## Materials and methods

### Samples

Canine samples used in this study were leftover lithium-heparinized plasma aliquots from routine diagnostic submissions to our laboratory from client-owned dogs with naturally occurring disorders. Therefore, ethical approval was waived by the animal welfare officers of the University of Bern and the cantonal veterinary office of Bern, Switzerland, but signed owner consent for the use of surplus biological samples and clinical data was available. Most samples were taken at admission to the small animal hospital, except where the clinical disease (notably sepsis) developed during hospitalization. Samples from clinically healthy dogs were surplus from a previously conducted reference interval study and from healthy controls of another clinical study, where owners agreed to participate by signing consent forms (Swiss Federal Food Safety and Veterinary Office approval nr. BE 127/19). Additionally, archived plasma from dogs enrolled in the hospital’s blood donor program were included. Lithium-heparinized whole blood was centrifuged at 6080 g for 2 minutes (Mikro 200, Hettich AG, Bäch, Switzerland) upon receipt in the laboratory and the supernatant plasma was stored in aliquots of 50 μl at -80°C pending analysis. Samples taken out of hours were considered eligible despite delayed processing and prolonged storage due to the robust stability of REG3E protein [23].

### Collected data

The electronic clinical and laboratory information databases were retrospectively searched for signalment, clinical diagnosis, vital parameters, survival to discharge, ultrasonographic findings, and results from additional tests including cytologic, histopathologic, and bacteriologic analyses. Recorded laboratory data included CRP concentrations and 1,2-o-dilauryl-rac-glycero-3-glutaric acid-(6’-methylresorufin) ester (DGGR)-lipase activity, both measured using a commercial biochemistry analyzer (Cobas c501, Roche Diagnostics, Basel, Switzerland). The CRP assay used was Randox canine CRP (Randox Laboratories Limited, Crumlin, UK) for analyses performed before January 1^st^ 2022 and Gentian canine CRP (Gentian AS, Moss, Norway) for analyses performed thereafter. Total leukocyte concentrations were measured with a commercial hematology analyzer using veterinary-specific software (ADVIA 2120i, Siemens Healthcare, Zurich, Switzerland) and band neutrophil percentages were obtained by manual differential cell counts on blood smears, performed by trained laboratory technicians.

### Animals

Control dogs were deemed healthy if there were no abnormalities reported by the owners, if the clinical examination revealed no abnormalities, and if there were no major abnormalities suggestive of a clinically relevant disease on their biochemistry and hematology profiles.

Inclusion criteria for the chronic GID group included clinical signs compatible with GID (i.e., vomiting, diarrhea, weight loss, inappetence) of >3 weeks duration and histopathologic evidence of enteritis or a strong clinical and ultrasonographic suspicion (e.g., altered mucosal echogenicity or thickening of one or more layers of the intestinal wall) with exclusion of other causes of diarrhea (such as metabolic, neoplastic, or infectious etiologies).

Samples were included in the acute GID group if dogs were presented for acute idiopathic vomiting and/or diarrhea of ≤1 week duration. Dogs with a clinical or laboratory suspicion of concurrent sepsis or AP, as described below, were excluded from this group.

The AP group included dogs which fulfilled at least 2 of the following criteria: DGGR-lipase activity at least 3x over the upper reference limit (URL), i.e., > 468 U/L, ultrasonographic suspicion of AP, and ≥2 clinical signs of anorexia, vomiting, diarrhea, and cranial abdominal pain. Dogs with suspicion of a pancreatic neoplasm based on clinical presentation, ultrasonographic or histopathologic findings, or a concurrent infectious process were excluded.

Dogs were included in the sepsis group if a bacterial infection in a body cavity or viscera was documented either through microscopic visualization of bacteria, positive culture result, macroscopic presence of pus, or overt leakage of intestinal contents into the peritoneal cavity visualized during explorative laparotomy. Where available, vital parameters at the time of blood sampling and concurrent leukogram data were recorded to assess criteria of SIRS. Cases were included if they fulfilled at least 2 of the 4 following [24]: temperature <100.6 or >102.6°F (<38.1 or >39.2°C), heart rate >120/min, respiratory rate >20/min, and white blood cells <6 or >16 x10^9^/L or >3% band neutrophils. Cases with a concurrent neoplastic disease were excluded.

### ELISA

Plasma samples were measured with an in-house developed sandwich ELISA, using custom-made polyclonal anti-canine REG3E antibodies raised in rabbits and guinea pigs against recombinant canine REG3E protein at 1:1000 dilution, as previously described [23]*.* This assay underwent extensive analytical validation and fulfills current international quality standards for laboratory testing, including specificity, precision, accuracy, linearity, parallelism, and spiking recovery, with robust sample stability and no significant interference by lipemia, hemolysis, or icterus [23]. Plates were processed using an automated reader (Crocodile 5-in-one ELISA miniWorkstation, Berthold Technologies GmbH & Co. KG, Bad Wildbach, Germany) and REG3E concentrations were calculated using a four-parameter logistic regression standard curve using commercial software (MicroWin 2010, Labsis Laborsysteme GmbH, Neunkirchen-Seelscheid, Germany). The measuring range of the assay covers concentrations from 30–300 ng/mL. Samples were applied in duplicates at 1:25, 1:250, 1:1000, or 1:5000 dilution in 1% bovine serum albumin in tris-buffered saline, depending on anticipated REG3E concentrations based on clinical presentation, with repeated measurements at higher or lower dilutions as necessary where measurements fell outside the measuring range. Where results were below the lower limit of quantification, they were recorded as <30 ng/mL and set as 30 ng/mL for statistical analysis. Control material from pooled canine plasma samples at three different levels were run alongside study dog samples. Coefficients of variation (CV) were calculated between duplicate measurements of all samples on the same plate as well as between control materials on different plates.

### Statistical analysis

Differences in demographic data among groups were assessed using Kruskal-Wallis tests for continuous variables and chi-squared tests for categorical data. Concentrations of REG3E among groups and between survivors and non-survivors across and within groups were compared using Kruskal-Wallis tests with post-hoc Conover analysis, independent samples t-tests where data were normally distributed after logarithmic transformation, or Mann-Whitney tests when normality was not met. Non-survivors included both dogs being euthanized and those naturally succumbing to their disease. Receiver operating characteristic (ROC) curve analysis with calculation of the Youden index (differential positive rate) was used to determine sensitivity, specificity, and positive and negative likelihood ratios of different REG3E concentrations to predict survival. Rank correlation was performed to assess the relationship between REG3E concentrations and DGGR-lipase activity or CRP concentrations. As our laboratory changed CRP reagents during the study period, only samples measured with the newer assay, as specified above, were included in the correlation analysis. Statistical analysis was performed using commercially available software (MedCalc® Statistical Software version 20.017, MedCalc Software Ltd, Ostend, Belgium; https://www.medcalc.org; 2023) and results were considered to be statistically significant if *P* ≤ .05, except for the post-hoc Conover analysis, where *P* < 0.005 was considered to be significant to account for multiple testing (Bonferroni correction for 10 pair-wise comparisons).

## Results

### Demographic data

A total of 214 dogs were enrolled, including 80 clinically healthy dogs, 23 and 25 dogs with chronic and acute GID, respectively, 42 dogs with AP, and 44 with sepsis. Demographic data of the five groups are summarized in Table 1. Age was significantly higher in the pancreatitis group and the sepsis group compared with all other groups (*P* < 0.005), with the pancreatitis group having the highest age across all groups.

**Table 1:** Demographic and laboratory data of 214 Dogs with and without different inflammatory diseases.

Variable	Healthy	Chronic GID	Acute GID	Acute Pancreatitis	Sepsis	*P* value
**n**	80	23	25	42	44	
**Age** [yrs], median (Min; IQR; Max)	4.5 (1; 2.1–6.8; 11.6)	4.6 (0.8; 2.2–7.1; 10.9)	2.9 (0.4; 1.9–5.6; 13)	11.3 (0.3; 8.2–12.8; 17.1)	8.4 (0.6; 6.1–10.4; 16.2)	< 0.001
**Sex**, n (%)						
Male; neutered Female; spayed	39 (49%); 15 41 (51%); 21	14 (61%); 4 9 (39%); 9	16 (64%); 6 9 (36%); 4	24 (57%); 19 18 (43%); 16	15 (34%); 9 29 (66%); 11	< 0.001
**Survivors** n (%)	80 (100%)	23 (100%)	25 (100%)	29 (69%)	24 (55%)	< 0.001
**REG3E** [ng/mL], median (Min; IQR; Max)	36.5 (<30; 30–89; 379)	200 (<30; 103.5–361.5; 1076)	634 (<30; 257–1060; 3626)	1644.5 (<30; 710–4122; 44699)	1736 (41; 480.5–3416; 40952)	< 0.001
**DGGR-Lipase** [U/L], median (Min; IQR; Max)	53 (12; 35–65; 156)	68 (28; 47.25–123.5; 278)	51 (13; 30.75–81.75; 177)	2470.5 (232; 1110–5285; 22400)	157.5 (13; 46.5–590; 2626)	< 0.001
**CRP**^a^ [mg/L], median (Min; IQR; Max) n	2.9 (0; 1.28–4.53; 21.9) 61	4.8 (0; 2.8–12.75; 45.9) 13	94.5 (10.6; 55.8–126.3; 265.5) 17	135.7 (3.9; 91.85–200.9; 415.4) 19	181.5 (11.4; 113.3–240.6; 605.8) 34	< 0.001

CRP, C-reactive protein; DGGR, 1,2-o-dilauryl-rac-glycero-3-glutaric acid-(6’-methylresorufin) ester; GID, gastrointestinal disease; IQR, interquartile range; REG3E, regenerating island-derived protein 3E.

^a^Only C-reactive protein results measured with the new assay are reported in this table.

There was a significant difference in breed and neuter status distribution among groups (*P* < 0.001), with female dogs, particularly entire bitches, being overrepresented in the sepsis group. Eighty-one different breeds were recorded, with mixed breed dogs being the most prevalent across the entire study cohort (14%) and within the healthy group (20%), the chronic GID (17%), and the acute GID group (16%). Other common breeds per group were: German Shepherd (9%) and Malinois (5%) in the healthy group; French Bulldog (17%) and Jack Russell Terrier (9%) in the chronic GID group; French Bulldog (8%), Jack Russell Terrier (8%), Labrador Retriever (8%), and Pomeranian (8%) in the acute GID group; Jack Russell Terrier (12%), Chihuahua (10%), mixed breed dog (10%), and Yorkshire Terrier (10%) in the AP group; Labrador Retriever (14%), American Staffordshire Terrier (7%), Border Collie (7%), and mixed breed dog (7%) in the sepsis group.

### Clinical and routine laboratory data

In the chronic GID group, biopsies of the gastrointestinal tract to establish the diagnosis of an inflammatory disease were performed in all except 2 cases. Immunosuppressant-responsive enteropathy was diagnosed in 10 dogs, including 8 with concurrent protein-losing enteropathy. Three dogs had colitis, one each food-responsive and non-responsive enteropathy, and in 8 dogs, the chronic enteropathy could not be further classified. DGGR-lipase activity was above reference intervals in 4 cases, none of which exceeded 3x the URL. Concentrations of CRP were measured in 21/23 dogs and were above reference intervals (>10.2 mg/L) in 7 (33%). All cases survived to discharge.

Most dogs (21/25) in the acute GID group had both acute diarrhea and vomiting, 3 dogs had only diarrhea, and 2 only vomiting. Activity of DGGR-lipase was minimally above the URL in 1 dog, and CRP was above reference intervals in all dogs. All cases survived to discharge.

In the AP group, 38/42 (90%) dogs had at least 2 of 4 suggestive clinical signs and an ultrasonographic suspicion of pancreatitis was present in 34/42 (81%). Activity of DGGR-lipase was above the URL (>156 U/L) in all dogs and above 3x the URL in 39/42 (93%). Concentrations of CRP were measured in 39 dogs and were above the URL in 30 (77%). Concurrent diseases were present in 32 dogs, including enteropathy (7), hyperadrenocorticism (7), hepatopathy (6), endocardiosis (5), renal disease (3 acute, 3 chronic, 1 glomerulonephritis), diabetic ketoacidosis (4), cholecystopathy (2), hypoadrenocorticism (2), and 1 each of trauma, benign prostatic hyperplasia, and idiopathic epilepsy, with several dogs having more than one co-morbidity. In total, 13/42 dogs with AP (31%) did not survive to discharge, one of which died of cardiovascular arrest, with the remaining 12 being euthanized primarily due to severity of disease and poor prognosis.

In the sepsis group, 40/44 (91%) dogs had sufficient data at the time of sampling to evaluate SIRS criteria; 2/4 criteria were met in 10 dogs (25%), 3/4 in 19 dogs (47.5%), and 4/4 in 11 dogs (27.5%). All except one dog with available hematological data (n = 42) had a left shift, and concurrent CRP was measured and above the URL in all dogs. Presence of bacteria was confirmed microscopically or by culture in 33 cases (75%). Septic peritonitis was diagnosed in 23 dogs (52%) and pyometra in 16 (36%), with both being present in 3 dogs. Other causes for sepsis included abscesses (5, of which 2 with concurrent septic peritonitis and 1 with septic meningitis), 3 wound infections, 1 septic retroperitonitis, and 1 septic arthritis with suspicion of septicemia. Of the 20/44 dogs (45%) that did not survive to discharge, half were euthanized on humanitarian grounds primarily due to severity of disease and poor prognosis, and the other half succumbed to their illness with cardiopulmonary arrest. The activity of DGGR-lipase was above the URL in 22 dogs (50%), and>3x URL in 13 dogs (30%).

### REG3E measurements

The mean CV between duplicates of all study dog samples on the same plate (intra-assay variability) was 5% (maximum 16%), and the mean CV between duplicates of control materials on the same plate was 5.6% (maximum 14.5%). The mean inter-assay CV for all three levels of control material across all plates was 11.2% (maximum 14.7%).

**Fig 1:**
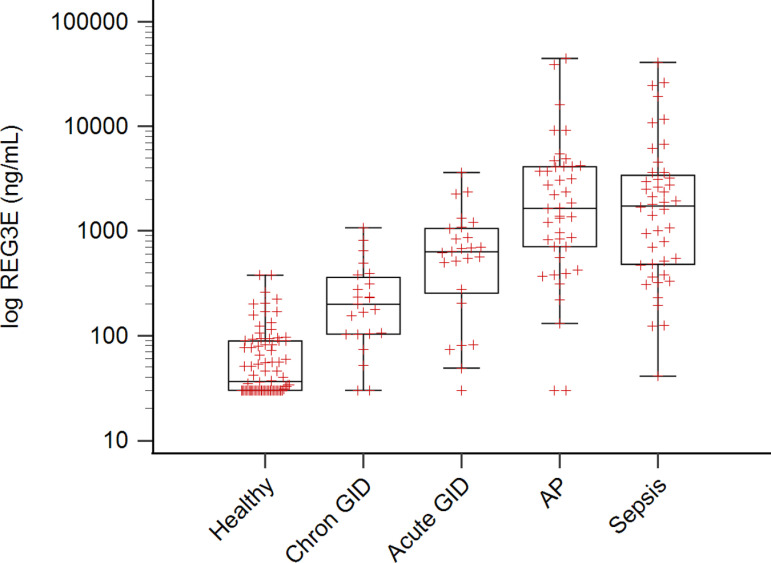
Box-and-whiskers plot comparing REG3E concentrations in dogs of different groups. Healthy, chronic gastrointestinal disease (GID), acute GID, acute pancreatitis (AP), and sepsis. There is a significant difference (*P* < 0.005) between all groups, except between the AP and sepsis group.

Healthy dogs had the lowest REG3E concentrations, followed by dogs in the chronic GID, acute GID, AP and sepsis groups (Table 1, Fig 1). REG3E concentrations in all diseased groups were significantly higher than in the healthy controls (*P* < 0.005), and there was a significant difference among all disease groups (*P* < 0.005), except between the AP and sepsis groups (*P* = 0.936).

**Fig 2:**
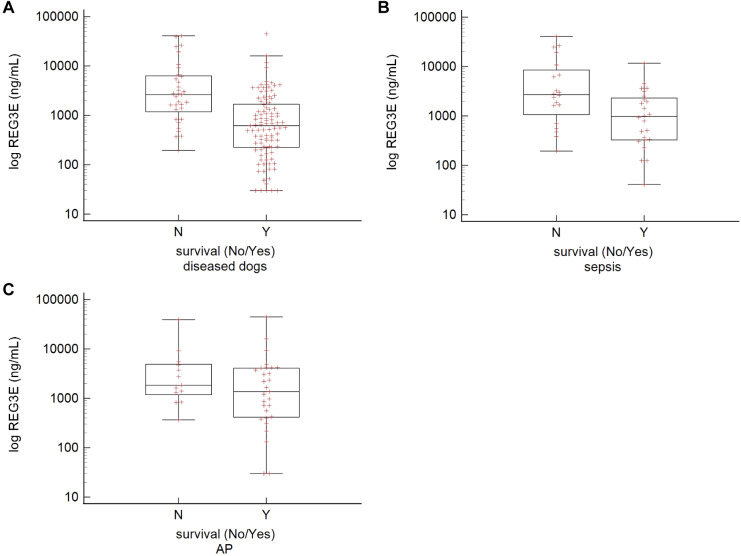
Box-and-whiskers plots of log REG3E concentrations of non-survivors and survivors. (A) all diseased dogs, (B) dogs with sepsis, (C) dogs with acute pancreatitis (AP). There is a significant difference between REG3E concentrations in survivors vs. non-survivors in all diseased dogs (*P* < 0.001) and septic dogs (*P* = 0.013) but not in dogs with AP.

**Fig 3:**
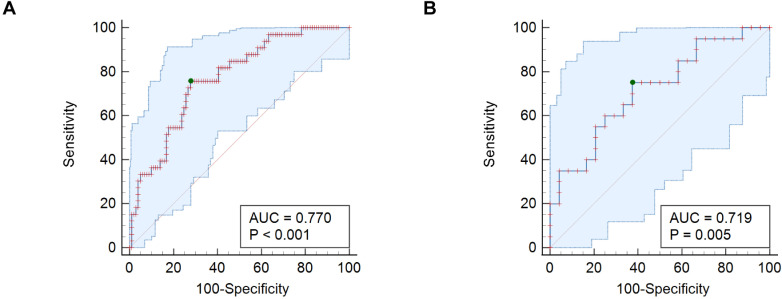
ROC curve analyses for prediction of non-survival with REG3E concentrations. (A) all diseased dogs, (B) dogs with sepsis only. Blue area, 95% confidence bounds for the ROC curve; Green dot, criterion for best cut-off calculated by the Youden index.

Concentrations of REG3E were significantly higher in non-survivors (median, 2622 ng/mL; IQR, 1207–6327.25 ng/mL) compared with survivors across diseased animals (*P* < 0.001; survivors median, 620 ng/mL; IQR, 226.5–1691.75 ng/mL) (Fig 2A). In dogs with sepsis, REG3E concentrations in survivors (median, 973.5 ng/mL; IQR, 327.5–2319 ng/mL) were significantly lower than in non-survivors (median, 2691 ng/mL; IQR, 1162–8764.5 ng/mL; *P* = 0.013). There was no difference between survivors (median, 1368 ng/mL; IQR, 416–4092 ng/mL) and non-survivors (median, 1847 ng/mL; IQR, 1207–4913.5 ng/mL) within the AP group (*P* = 0.247; Figs 2B and 2C). The AUC (area under the curve) of the ROC curve for REG3E measurements to predict non-survival in all clinically ill dogs was 0.77 (95% CI, 0.69–0.839; *P* < 0.001) with a cut-off of >1330 ng/mL (Fig 3A). When assessing only dogs with sepsis, the AUC was 0.72 (95% CI, 0.563–0.844; *P* = 0.005) with a cut-off of >1414 ng/mL (Fig 3B). Dogs with REG3E concentrations above these cut-offs were at least twice as likely to die than dogs with lower REG3E concentrations. Sensitivities, specificities, and likelihood ratios corresponding to these cut-offs are given in Table 2.

**Table 2:** Optimal REG3E cutoffs, as calculated by ROC curve analysis and the Youden index, and associated criteria, for prediction of non-survival.

Group	Cut-off [ng/mL] (95% CI)	Sensitivity % (95% CI)	Specificity % (95% CI)	+ LR (95% CI)	- LR (95% CI)
**Diseased dogs**	1330 (686–4545)	75.76 (58–89)	72.28 (63–81)	2.73 (1.89–3.95)	0.34 (0.18–0.62)
**Sepsis**	1414 (324–4545)	75 (51–91)	62.5 (41–81)	2.0 (1.13–3.55)	0.4 (0.18–0.91)

CI, confidence interval; + LR, positive likelihood ratio; -LR, negative likelihood ratio

The correlation between REG3E concentrations and DGGR-lipase activity was low when including all samples (Kendall’s Tau = 0.344; 95% CI, 0.248–0.431; *P* < 0.001) as well as within the AP group (Kendall’s Tau = 0.365; 95% CI, 0.176–0.39; *P* < 0.001); there was no correlation between REG3E concentrations and DGGR-lipase activity within any of the other groups (*P* > 0.313). The correlation between REG3E and CRP concentrations was moderate when calculated across all samples with CRP measured by the new assay (n = 144; Kendall’s Tau = 0.555; 95% CI, 0.476–0.613; *P* < 0.001) and low within the sepsis group (Kendall’s Tau = 0.28; 95% CI, 0.081–0.466; *P* = 0.0207). There was no correlation between CRP and REG3E concentrations in any of the other groups (*P* > 0.0765).

## Discussion

In this study we demonstrate that REG3E concentrations were higher in dogs with sepsis, AP, and, to lesser degree, GID, compared with concentrations in healthy dogs. This parallels findings in humans, where patients with inflammatory bowel disease have only mildly higher REG3A concentrations, whereas patients with AP and sepsis often have markedly higher REG3A and REG1A concentrations in comparison with healthy volunteers [18,25,26]. However, our results cannot be directly compared to results in humans, as we are measuring different proteins using different assays, with disease definitions, clinical presentations, and diagnostic workups differing substantially between human and veterinary medicine.

We found markedly higher REG3E concentrations in dogs with sepsis and AP compared with our healthy control group, but the magnitude in both diseased groups was similar. Therefore, we conclude that REG3E cannot discriminate between these diseases, and is by extension, in contrast to REG1A in humans [26,27], unlikely to be able to differentiate between sepsis and non-septic SIRS. We have excluded cases for which there was a suspicion of concurrent pancreatitis and sepsis, as well as dogs affected by suspected confounders such as pancreatic and intestinal neoplasia in an attempt to keep our groups as distinct as possible. Nonetheless, severe AP can cause non-septic SIRS and culminate in organ failure, therefore, there was likely an overlap in the degree of systemic inflammation between the AP and the sepsis groups. Furthermore, AP can arise as a complication of critical illness in humans [28] and, with our current diagnostic methods, it was not possible to completely exclude concurrent AP in all cases, particularly in already severely ill dogs. Hyperlipasemia in absence of overt pancreatitis is not uncommon in critically ill patients, both in humans and dogs [29,30], thus it is possible that pancreatic damage is more common than clinically suspected in dogs with SIRS or sepsis.

Although dogs with chronic GID had a significantly higher median REG3E concentration compared with controls, there was considerable overlap, with approximately three-quarters of dogs with chronic GID having REG3E concentrations within the range measured for healthy control dogs. Similar findings have been reported in humans, where serum REG3A has poor accuracy for the diagnosis of active inflammatory bowel disease (IBD) [31]. In a comparative proteomic study using canine feces, dogs with chronic GID had higher concentrations of a REG3A/PAP homolog, which is likely the same protein as REG3E [21], than control dogs, but there was also some overlap, particularly between dogs with food-responsive enteropathies and healthy dogs [22]. However, protein concentrations in feces may not accurately mirror those measured in blood as studies in humans with IBD have shown that serum and fecal concentrations of REG3A correlate only weakly [31]. This may be attributed to REG production in other tissues, discrepancies between secretion of proteins into the gastrointestinal lumen and reabsorption into circulation, as well as intermittent secretion into feces leading to great intra-individual variability between bowel movements. Nonetheless, based on the currently available data, it is unlikely that REG3E is a reliable biomarker for chronic enteropathies in dogs.

Dogs with acute GID had significantly higher REG3E concentrations compared with healthy dogs and those with chronic GID, and significantly lower values compared with AP and sepsis. We deliberately excluded dogs with a clinical concern for bacterial translocation or other suspicion of sepsis from the acute GID group, as this would have blurred the distinction from dogs with sepsis. This has likely resulted in a selection of less severe acute GID cases, and REG3E concentrations in dogs with acute hemorrhagic diarrhea syndrome and bacterial translocation may have values comparable to the septic group. Nonetheless, we believe that these results provide some insight, particularly when compared with the chronic GID group, as this could suggest that REG3E mirrors the degree of inflammation rather than the gastrointestinal origin of disease in these cases. There was considerable overlap of REG3E concentrations between dogs with acute GID and AP, thus the clinical usefulness of REG3E to distinguish these two diseases with potentially very similar clinical pictures is questionable. Nonetheless, very high REG3E concentrations were more likely to be seen with AP in our cohort, the significance of which remains to be explored in larger studies.

The REG protein family has previously been designated as “stress proteins” [32,33] and rat REG3A was proposed to be a class 2 acute phase protein owing to its stimulation by IL-6 in combination with glucocorticoids [34]. Per definition, major acute phase proteins have very low baseline concentrations in blood, which increase at least 100–1000 fold upon inflammatory stimulation, with a peak within the first 1–2 days and a rapid normalization during recovery [35]. In rodents, REG3 protein expression in pancreatic tissue increase up to 5000-fold within 24 hours of induced pancreatic damage followed by a rapid decrease over 2–3 days after resolution of inflammation [32]. Thus, if the kinetics of canine REG3E in blood follow a similar pattern as observed for REG3A in other species, then based on our findings of markedly higher concentrations of REG3E in different inflammatory diseases, this protein can be considered a major positive acute phase reactant in dogs.

Despite these findings, the correlation with CRP, one of the most widely used positive acute phase proteins in dogs, was only low to moderate in our study. Possible explanations for this discrepancy include different kinetics of REG3E and CRP, peaking at different times in the course of inflammation, or triggers other than inflammation contributing to high REG3E concentrations. In humans, renal disease is also associated with higher REG1A concentrations, with values exceeding those of patients with acute or chronic pancreatitis within the same study [36,37]. In our cohort, 7 dogs with pancreatitis had concurrent renal disease, including acute, chronic, and glomerular disease, which could have affected REG concentrations. Interestingly, 6/7 dogs with concurrent renal disease had REG3E concentrations above the 75^th^ percentile of all AP cases, including the case with the highest measured REG3E concentrations in the entire cohort. In humans, REG concentrations have also been found to be high with renal failure [36,37]. Therefore, it is plausible that renal disease could lead to an increase in REG3E concentrations in dogs, but underlying pathomechanisms remain to date unexplored.

Similarly, REG3E concentrations correlated poorly with DGGR-lipase activity, the main marker for pancreatic injury used in our laboratory for dogs. This could mirror extrapancreatic origin of the protein or upregulation in response to inflammation remote from the pancreas, as was demonstrated for REG3 proteins in rats [12].

We could demonstrate that REG3E concentrations differed significantly between survivors and non-survivors when taken across all diseased dogs and within the sepsis group, but not between survivors and non-survivors with AP. Septic dogs with a REG3E concentration above 1414 ng/mL were twice as likely to die or be euthanized than septic dogs with REG3E concentrations below this threshold, but sensitivity and specificity were not high enough to advocate the use of REG3E as the sole predictor of outcome in our study. In contrast, REG1A is considered a reliable prognostic indicator in studies in humans [38,39], but survival cannot be directly compared between studies in humans and dogs. On one hand, euthanasia of veterinary patients can be elected for several reasons, including financial and ethical, and does thus not always reflect the severity of the disease. On the other hand, the standard of care in veterinary medicine still lags behind compared to human medicine, which can result in higher non-survival in face of similar disease severity. Furthermore, it is also possible that the peak REG concentration was missed in some dogs in our study, as blood was not sampled on each consecutive day and in some cases only at admission. Serial measurements of REG3A in humans with AP demonstrated that in most patients a maximum concentration is reached during the first few days of hospitalization rather than at admission [18], but further research is needed to establish whether kinetics of canine REG3E parallel those of REG3A in humans with AP.

Our study had multiple shortcomings in addition to the limitations addressed above. One being the retrospective rather than prospective case enrollment, which would have allowed for more uniform study cohorts and consistent sampling times. Many of our dogs with pancreatitis had concurrent diseases, some of which, such as gastroenteropathies, renal and hepatic disease, or diabetes mellitus, may have contributed to the high REG3E concentrations in this group, as reported for REG1A in humans [36,37]. Furthermore, no *a priori* power analysis could be performed because of the lack of available data on expected REG3E concentrations in dogs. Nonetheless, given the substantial differences found between our healthy and diseased dogs, it is unlikely that our study was underpowered. We based our definition of SIRS on criteria extrapolated from the human definition [24], which, although still widely used in veterinary medicine, has poor specificity in small animals [40]. Therefore, this may have biased the study cohort towards less severe disease, which could have underestimated REG3E concentrations in this group of dogs. Additionally, it is unknown how REG3E concentrations develop with increasing age in dogs, therefore we cannot exclude that the higher age of dogs in the sepsis and AP group compared to all other cohorts may have influenced REG3E concentrations. In humans, it is well established that normal REG1A concentrations vary with age, with the lowest values measured in neonates and the highest in children [41]. In our study, 7 dogs (at least one from each disease category) were under one year of age, which may have potentially positively skewed their REG3E concentrations, particularly as all healthy dogs were over 1 year old. Future prospective studies investigating REG3E should ideally age-match cases and controls.

## Conclusion

In conclusion, we measured high REG3E concentrations in dogs with AP and sepsis, and to lesser degree with acute and chronic GID, albeit with substantial overlap of REG3E concentrations among different diseases. Thus, REG3E is more likely to reflect the severity of inflammation than the underlying condition. As such, REG3E is more likely to represent a general positive acute phase protein in this species rather than a diagnostic aid for gastrointestinal or pancreatic disease, or for the differentiation of sepsis from non-septic SIRS. Nonetheless, the potential of REG3E as a useful biomarker as part of a panel of laboratory parameters with integration of clinical data to distinguish between different diseases and to potentially guide decision-making in a clinical setting deserves further investigation.
